# Direct health system costs for systemic lupus erythematosus patients in Alberta, Canada

**DOI:** 10.1371/journal.pone.0251409

**Published:** 2021-05-07

**Authors:** Francis Fatoye, Tadesse Gebrye, Lawrence W. Svenson

**Affiliations:** 1 Department of Health Professions, Manchester Metropolitan University, Manchester, United Kingdom; 2 Analytics and Performance Reporting Branch, Alberta Health, Edmonton, Canada; 3 Division of Preventive Medicine, University of Alberta, Edmonton, Canada; 4 School of Public Health, University of Alberta, Edmonton, Canada; 5 Department of Community Health Sciences, University of Calgary, Calgary, Canada; Universita degli Studi dell’Insubria, ITALY

## Abstract

Systemic lupus Erythematosus (SLE) is a chronic multi-system autoimmune disease that can affect a person’s physical, mental, and social life. It imposes a substantial economic burden up on patients, carers, healthcare systems, and wider society. This is the first study to examine the direct health care costs of SLE in Alberta using real-world data. Alberta maintains a publicly funded, universally available health care system. Health service use and direct healthcare costs of SLE and non-SLE cases were determined from inpatient hospital services, fee-for-physician services, emergency services, and ambulatory care services. All costs were estimated for calendar year 2016. Data were analysed using central measures specifically the mean to determine the annual costs of SLE and non-SLE. A total number of 10,932 (Male = 2,546; Female = 8,386), and 41,851,36 (Male = 21,157,76; Female = 20,693,60) of SLE and non-SLE cases, respectively were included in this study. The mean annual costs of SLE, and non-SLE per case were $7,740.19 (Male = $7,986.59; Female = $7,665.38), and $2,479.53 (Male = $2,265.57; Female = $2,698.30), (p < 0.001) respectively. The mean annual costs of fee-for-physician services (SLE = $2,160.03; non-SLE = $840.00) (p < 0.001), inpatient hospital services (SLE = $3,462.86; non-SLE = $1,007.29), (p < 0.001) emergency services (SLE = $440.28; non-SLE = $176.65), (p < 0.001) and ambulatory care services (SLE = $1,677.03; non-SLE = $455.05) (p < 0.001) per case were estimated. The findings showed that the costs of SLE were considerably high for patients and healthcare system. This highlights the importance of appropriate treatment and management of SLE. Further studies are required to fully investigate both the direct and indirect economic burden of SLE including out-of-pocket expenses, costs to patients and caregivers and productivity loss.

## Introduction

Systemic lupus erythematosus (SLE) is a chronic systemic autoimmune disease that affects multiple organ systems in the body, and is a cause of physical and functional disability [1]. Although SLE is an extremely heterogeneous disease in terms of progression or development, its prevalence, incidence, and socioeconomic impact is substantial. Evidence from the Michigan Lupus Epidemiology and Surveillance Programme suggested a prevalence of 72.8 and incidence of 5.5 SLE per 100,000 population per year [2]. Other studies have also reported the incidence of SLE of 1.0 per 100,000 per year in Denmark [3]; and 4.43 per 1,000,000 in Alberta [4]. Environmental factors contributing to the development of SLE include cigarette smoking, and exposure to silica, pollution, and/or solvents [5]. The severe manifestation and unpredictable experience of patients with SLE may result to substantial healthcare resource utilization. This is associated with fee for physician visits and other healthcare professionals, diagnostic tests, procedures, hospitalizations, medications, and costs that result from loss of employment and diminished work productivity [6].

The diverse manifestation of SLE and its treatment may incur a substantial direct and indirect costs to patients with these conditions. For example, the total direct costs of SLE per patient per year in Taiwan [7], South Korea [8], Germany [9], and the United States of America (USA) [10] were estimated as $1,847, $4,940, $4,887, and $5,457, respectively. The annual indirect costs of SLE per patient have been estimated in the United Kingdom [11], Sweden [12], and Germany [9] as $12,309, $20,046, and $17,184, respectively. The mean annual direct costs estimate of SLE per patient in Canada was $5,062 [13]. Of this annual direct costs of SLE in Canada, the contribution of health professional visit, emergency room visit, diagnostic procedure, outpatient surgery, and inpatient care was $893, $1,058, $605, $126, and $2,404, respectively.

The previous published study of the costs of SLE in Canada estimated the costs of SLE via questionnaire survey with limited sample size. The use of questionnaire can either over or underestimate the costs associated with SLE and as such may not be generalizable in the real-world. In addition, no previous studies have compared the costs of SLE with those of non-SLE case. This study examined the direct health care costs of SLE, compared to non-SLE cases for inpatient hospital services, fee-for-physician services, emergency department services, and ambulatory care services in the real-world in Canada. Studies conducted using real-world data from a variety of sources such as electronic health records, claims and billing activities, claims and billing data, and disease registries are believed to solve the problem of generalizability.

## Materials and methods

The province of Alberta, Canada maintains a publicly funded, universally available healthcare system. All residents of the province are required, by law, to register with the Alberta Health Care Insurance Plan, which is estimated to cover over 99% of the population. Each person is assigned a personal health number that acts as a unique lifetime identifier (ULI) that is captured during health system encounters. This allows for deterministic linkage of data at the individual level, across the basket of services available.

The data used in this study were released in aggregate from the Ministry of Health of Alberta, and it’s use is covered by s. 26, meaning formal ethics was not required to access the data. We used routinely collected data, analysed anonymously, and individual patient consent was not required.

Alberta has a population-based, province-wide, discharge abstracts database which includes all inpatient hospitalizations, ambulatory (outpatient) hospital visits, and emergency department visits for patients of all ages. The province also maintains a comprehensive physician claims database which captures all physician visits, regardless of service location or setting. Diagnosis was extracted from the ICD 9 or ICD 10 codes provided as part of a hospital (inpatient, outpatient, and emergency department) or physician visit. In order to do this an algorithm was applied and this is described elsewhere [14, 15]. Bernatsky and colleagues showed that the data can be used, when an algorithm is applied, to improve specificity [15]. What this means for this study is that those we classified have a high probability of being accurately assigned. The lower sensitivity suggests we are underestimating the number of cases. In practice this means we are erring on misclassifying cases as non-cases, so are providing conservative estimates of the cost differential. However, there was no specific information available on the administrative data that speaks to which tests were used during a specific visit. Physician may code up to three diagnoses per encounter, while up to 25 are captured for inpatient stays, and up to 10 for ambulatory or emergency department services. Individuals were classified as having SLE if they had at least three physician services over a two year period, with a minimum of 60 days between the first and second services, or one or more hospitalizations [4, 16].

Health care resource use and direct healthcare costs of SLE and non-SLE cases were determined from inpatient hospital services, fee-for-physician services, emergency department services, and ambulatory care services. All costs were estimated for calendar year or financial year of 2016, and were estimated at the ULI level. The net fee paid to physicians was tabulated directly from adjudicated physician claims data. Because patient-event level case costing is only available for a portion of inpatient, ambulatory, and emergency visits, groupers were used to estimate the cost of these events. For ambulatory and emergency visits, the Comprehensive Ambulatory Classification System (CACS) grouper, developed by the Canadian Institute for Health Information (CIHI), was used to link a Resource Intensity Weight (RIW) to each event. The Cost per Weighted Case (CPWC, estimated with available case costing data) is applied to RIW values to estimate the cost associated with each event. A similar process was used to estimate the costs of inpatient events, using the RIW from the CIHI Case Mix Grouper Plus (CMG+) methodology, and the costs of a Standard Hospital Stay (CSHS). Inpatient cost estimates were pro-rated on an average per diem basis for events that began before Jan 1, 2016 or ended after December 31, 2016.

Descriptive statistics were used to describe the characteristics of cases with SLE and non-SLE. Summary statistics such as percentages and mean were presented.

## Results

The demographic characteristics of the participants included in this study are summarized (Table 1). A total number of 10,932 (Male = 2,546; Female = 8,386), and 4,185,136 (Male = 2,115,776; Female = 2,069,360) of SLE and non-SLE cases, respectively were included in this study. Of these, 328 (Male = 124; Female = 204) and 1,038,088 (Male = 530,666, Female = 507,422) cases of SLE and non SLE, respectively were below the age of 20 years. The highest and lowest percentages of SLE cases included in this study were patients ≥ 60 years and < 20 years of age, respectively. For both male and female participants, the number of cases of SLE increased with age.

**Table 1:** Characteristics of the participants.

Age (years)	Number of cases with SLE				Number of cases without SLE			
	Male	Female	All	%	Male	Female	All	%
**< 20**	124	204	**328**	3.0	530,666	507,421	**1,038,088**	24.8
**20–39**	413	1,462	**1,876**	17.0	662,974	629,384	**1,292,358**	30.8
**40–59**	952	3,380	**4,333**	39.6	575,256	552,726	**1,127,982**	27.0
**≥ 60**	1,057	3,339	**4,396**	40.0	346,880	379,829	**726,708**	17.4
**Total**	**2,546**	**8,386**	***10*,*932***		**2,115,776**	**2,069,360**	***4*,*185*,*136***	

The mean (SD) costs of SLE, and non-SLE per case are presented in Tables 2 and 3. The mean (SD) costs of SLE, and non-SLE per case were $7,981 (4,499) (Male = $7,192 (3,931); Female = $8,770(4989)), and $3,590 (3,204) (Male = $3,905 (3,647); Female = $3,276 (2761)), respectively. The mean costs of SLE per case were $4,391 higher than the non-SLE. The mean (SD) costs of fee-for-physician services (SLE = $1,960 (770); non-SLE = $1,069 (678)), inpatient hospital services (SLE = $3,911 (3338); non-SLE = $1,721 (2130)), emergency services (SLE = $472 (226); non-SLE = $220 (131)), and ambulatory care services (SLE = $1,637 (595); non-SLE = $580 (380)) per case were estimated. The key cost drivers for the direct health care costs of SLE were inpatient hospital services (49%).

**Table 2:** Mean costs for SLE and Non SLE per case.

Variable	SLE (Can$), Mean (SD)	Non SLE (Can$), Mean (SD)	P value
**MD**	1,960 (770)	1,069 (678)	< 0.001
**INPT**	3,911 (3338)	1,721 (2130)	< 0.001
**EMRG**	472 (226)	220 (131)	< 0.001
**AMB**	1,637 (595)	580 (380)	< 0.001
**Total**	**7,981 (4,499)**	**3,590 (3,204)**	**< 0.001**

MD = Fee-for-physician services; INPT = Inpatient hospital services; EMRG = Costs attributed to emergency department services; AMB = Ambulatory care services, or outpatient services provided by an acute care facility

**Table 3:** Costs of SLE and non SLE per case by sex.

Variable	Male				Female			
	SLE (Can$), Mean (SD)	%	Non SLE (Can$), Mean (SD)	%	SLE (Can$), Mean (SD)	%	Non SLE (Can$), Mean (SD)	%
**MD**	1,897 (790)	26	1,124 (727)	29	2,024 (766)	23	1,014 (642)	31
**INPT**	3,264 (2,802)	46	1,949 (2,529)	50	4,558 (3,768)	52	1,492 (1,683)	46
**EMRG**	452 (181)	6	233 (156)	6	492 (268)	6	207 (105)	6
**AMB**	1,579 (565)	22	598 (376)	15	1,694 (634)	19	561 (393)	17
**Total**	**7,192 (3,931)**		**3,905 (3,647)**		**8,770 (4,989)**		**3,276 (2,761)**	

MD = Fee-for- physician services; INPT = Inpatient hospital services; EMRG = Costs attributed to emergency department services; AMB = Ambulatory care services, or outpatient services provided by an acute care facility.

The annual costs of SLE and non-SLE per cases and non-cases by age group and service of care are presented in Table 4. For both cases of SLE and non-SLE, the annual costs incurred for fee-for physician services, inpatient hospital services, emergency department services and ambulatory care services increased with age. Those ≥ 60 years of age contributed the highest percentages of costs that is fee-for physician services (48%); inpatients hospital services (58%), emergency department services (46%) and ambulatory services (44%) due to SLE. Although costs incurred by each services of care increased with age in both SLE and non-SLE, the share of those ≥ 60 years of age to the total costs was higher for the SLE compared to non-SLE.

**Table 4:** Annual costs of SLE and Non SLE per total number of cases/individual case by age group and service of care.

Variable	SLE (Can$)/N (%)	SLE (Can$)/individual case	Non SLE (Can$)/N (%)	Non SLE (Can$)/individual case
**MD**				
**< 20**	344,965.75 (1)	1053.14	443,156,046.36 (13)	426.90
**20–39**	3,343,537.16 (14)	1782.50	859,841,017.00 (24)	665.33
**40–59**	8,585,826.06 (36)	1981.71	993,070,933.62 (28)	880.40
**≥ 60**	11,338,894.81(48)	2579.33	1,221,715,952.75 (35)	1681.16
**Total**	**23,613,223.78**	**7396.68**	**3,517,783,949.73**	**3653.78**
**INPT**				
**< 20**	533,498.89 (1)	1628.71	436,269,206.92 (10)	420.26
**20–39**	4,123,147.99 (11)	2198.12	678,043,613.05 (16)	524.66
**40–59**	11,058,824.3 (29)	2552.51	878,414,123.53 (21)	778.75
**≥ 60**	22,140,252.6 (58)	5036.38	2,222,935,530.2 (53)	3058.91
**Total**	**37,855,723.80**	**11415.72**	**4,215,662,473.78**	**4782.58**
**EMRG**				
**< 20**	123,805.86 (3)	377.97	138,339,975.1 (19)	133.26
**20–39**	837,429.99 (17)	446.45	205,707,030.6 (28)	159.17
**40–59**	1,618,961.26 (34)	373.68	180,600,280.5 (24)	160.11
**≥ 60**	2,232,862.6 (46)	507.92	214,659,171.3 (29)	295.39
**Total**	**4,813,059.69**	**1706.01**	**739,306,457.55**	**747.93**
**AMB**				
**< 20**	422,155.34 (2)	1288.79	281,730,978.15 (15)	271.39
**20–39**	2,928,289.5 (16)	1561.12	358,457,164.2 (19)	277.37
**40–59**	6,988,613.5 (38)	1613.06	546,915,349.08 (29)	484.86
**≥ 60**	7,994,084.13 (44)	1818.46	717,327,631.2 (38)	987.09
**Total**	**18,333,142.45**	** 6281.43**	**1,904,431,122.69**	** 2020.71**

MD = Fee-for- physician services; INPT = Inpatient hospital services; EMRG = Costs attributed to emergency department services; AMB = Ambulatory care services, or outpatient services provided by an acute care facility; N = Total number of cases.

The annual costs of SLE per total number of cases were estimated $84,615,150. Of this total costs, fee-for physician services, inpatient services, emergency department services, and ambulatory care services were $23,613,224, $37,855,724, $4,813,060, and $18,333,143, respectively. Overall, as people age, they are more likely to be hospitalized and that is the highest cost driver for SLE in this study.

## Discussion

We have provided estimates for direct health care costs associated with SLE and non-SLE in a publicly funded, single payer system in the province of Alberta, Canada using real-world data. The costs of SLE and non-SLE were estimated from a publicly funded and universally available health care system in Alberta. The findings of this study suggest that, the mean (SD) costs estimate of SLE per year was Can$7,981 (4,499) which are two times higher than those of non-SLE. The mean costs per case of fee-for physician services, inpatient hospital services, emergency department services, and ambulatory care services of SLE were 1.8, 2.3, 2.1, and 2.8 fold greater compared to those of non-SLE, respectively. Moreover, the annual costs of SLE per total number of cases in Alberta were estimated at Can$84,615,150.

Further, the findings of our study showed that the highest costs among the SLE case were in older age group. The reason for this is that people with SLE could stay more expensive throughout their life course due to related comorbidities [17]. This is consistent with Kang et al. who suggested the presence of comorbidities such as nephropathy in SLE patients had significantly higher total expenses for hospitalization during the end of life [18]. As people age, they are more likely to be hospitalized and that was the highest cost driver in the system. Therefore, even if SLE wasn’t causing a lot of grief, it could lead to a slightly longer hospital stay and therefore increase healthcare costs with age. The increased costs in the older population is more likely a function of an increased risk of hospitalization, rather than something specific to SLE. That said, that fact that our SLE cohort are more expensive at the higher ages suggest that SLE could increase the risk of comorbidity, length of stay in hospital, or the likelihood of being hospitalized for some other condition.

The findings of the current study were compared with previous studies that examined the direct costs of SLE [13, 19, 10, 9, 11] and other types of disease such as rheumatoid arthritis [20] in Canada and other countries. The results of the current study are very similar to those of Clarke and colleagues (Can$7,832.0) that have used 164 patients with SLE in Canada [13]. The direct cost in this study contained all resources utilized in patients’ care including ambulatory care, hospital care, and rehabilitation facility care. Another study that estimated the direct costs from 231 patients of SLE in Canada claimed that the mean annual direct costs of SLE was Can$4968.0 [10]. A review of 24 studies from eight different countries including Canada estimated the direct costs of rheumatoid arthritis (RA) per patient per year between $3,400 and $21,000 [20]. The estimate of the direct costs SLE in the current study was twice of the lowest estimate of RA. Parallel to this, the cost associated with inpatient care was the highest contributor of direct costs in both SLE (46%) and RA (75%) [20].

The direct costs estimate of SLE of the current study have shown a substantial variation compared to China (US$8,230.0), USA (US$13,305.0), and Germany (€3,191.0) and the United Kingdom (£2,613.0) [19, 20, 9, 11]. The direct cost estimate of SLE in the USA [21] were approximately 50% higher than the cost estimates in our study. One of the explaining factor for this could be the patients included in the USA’s study, they were from US commercial insurance claims database and were required to have ≥ 2 claims and should have continuous health plan enrolment for six months. Our direct cost estimates were 2 times higher than those in the United Kingdom after making correction to currency and inflation adjustment [11]. Overall, the methodologies used to conduct the studies including the types of resource used and the differences in the fee charged for healthcare in different countries may be the sources of variation for the direct cost estimates due to SLE.

The healthcare utilization estimates in our study were also compared with previous studies [13, 9, 19, 10, 21]. The costs estimate inpatient hospital service in our study were 1.3, 2.0, and 1.1 times of the Clarke et al. [13], Huscher et al. [9], and Pelletier et al. [20], respectively. The cost estimates of the emergency department service in Alberta have been shown to be 3.6 fold of the UK, and approximately identical to USA [11, 10]. The fee-for-physician services cost estimate in the current study was found to be approximately 50% lower in the USA [10, 21].

One of the strengths of this study was that it has used a large number of cases with SLE and non-SLE to determine the mean annual direct costs. This has enabled the researchers to estimate the costs of the healthcare utilization including fee-for-physician services, inpatient hospital services, emergency department services, and ambulatory care services from comprehensive information. Compared with the cost estimates derived from surveys, the direct costs estimate in our study is more reliable and generalizable. On the other hand, the present study may have inadequately identified all patients with SLE in Alberta, this may be due to the huge difference of the prevalence of SLE observed between the 2015 and 2016 calendar years [4]. Another limitation of this study was that we did not cost the prescription of drugs; this may underestimate the difference between those with SLE and those without. Overall, the authors believe that the annual direct cost estimate of SLE per case may have been underestimated due to the inadequate identification of patients with SLE.

## Conclusion

This is the first study to examine the direct health care costs of SLE, compared to non-SLE cases in the real-world. Individuals with SLE, when compared to those without, incurred, on average, three times greater direct health system costs. These findings highlight the importance of appropriate treatment and management to improve the health outcomes of patients with SLE and reduce the healthcare utilization associated with the management of the condition. Additional work is warranted to examine the overall costs of managing SLE and the specific cost drivers which may be avoidable, or better managed, in the future. For example, do SLE patients become more costly as they age and is this increase at a rate of growth that is different from the natural aging of individuals with non-SLE?.

## Supporting information

S1 Data (XLSX)
